# The influence of co-residential and non-co-residential living arrangements on sufficient fruit and vegetable consumption in the aging population in Thailand

**DOI:** 10.1186/s12877-020-01884-2

**Published:** 2020-11-16

**Authors:** Sirinya Phulkerd, Rossarin Soottipong Gray, Aphichat Chamratrithirong

**Affiliations:** Institute for Population and Social Research, Mahidol University, Nakhon Pathom, 73170 Thailand

**Keywords:** Older adults, Aging, Fruit and vegetable consumption, Living arrangements, Co-residence, Family structure, Household types

## Abstract

**Background:**

Living arrangements have an impact on a family’s health-related behaviors, especially its eating behaviors. However, studies that have examined the association between living arrangements and food intake, especially fruit and vegetable (FV) consumption of older adults, are rare. This study aimed to investigate the association between living arrangements and FV consumption in a population of older adults in Thailand from a national sample of households.

**Methods:**

This study extracted data on 2048 persons age 60 years or older from a study of a nationally-representative sample of Thai households. The survey asked respondents about FV intake, living arrangements, household size, and socio-demographic characteristics. Binary logistic regression analysis was used to investigate the association between the variables and FV intake.

**Results:**

The mean age of the respondents was 68.2 ± 6.5 years. Of the total sample, only 31.9% had sufficient FV intake. The group with the lowest possibility of sufficient FV intake was persons who lived alone. Those who lived with at least one child or lived in a skipped-generation household were 2.7 and 2.2 times as likely to have sufficient FV intake as those who lived alone (*p* < 0.001 and *p* < 0.01, respectively). Older adults living only with their spouse were 2.1 times as likely to have sufficient FV intake as those who lived alone. FV intake also differed significantly by socio-demographic characteristics (sex, place of residence, educational attainment, occupation and income), self-rated health, FV knowledge, and exposure to a FV promotion campaign in the community.

**Conclusions:**

The findings from this study suggest that a different approach is required to improve FV consumption in the older population by taking into account their living arrangements, community context, level of FV knowledge, and socio-demographic characteristics. The older adults who live alone, as well as those living in a large household, are at particular risk of inadequate FV intake, and require special attention.

## Background

All countries in Asia are undergoing profound and rapid population change. They are experiencing the increasing share of older adults in the population. This affects historical living arrangements, and thus brings more attention of the living arrangements of older adults to health and non-heath policy makers.

Family structure or household type plays an important role in shaping dietary intake of the household. There is extensive evidence of the association between the family structure, eating habits, and health status of household members. Several studies have found that children in single-adult-headed families, reconstituted families, or families without parents were more likely to have unhealthy eating habits, such as irregular breakfast consumption, eating less vegetables, and drinking alcohol, compared to children living with both parents [1–5]. However, the association between family structure and food intake of older adults has been little studied. A few studies show living alone to be associated with higher nutrition risk in older adults [6, 7]; another study found that home-living older adults with a female spouse was linked to good and nutritious food consumption [8]. Most prior research has investigated the association of living arrangements either with children’s diet and health [1–5, 9] or with older person’s health and well-being [1, 10–14]. Thus, examining influence of family structure on healthy diets -- especially sufficient fruit and vegetable (FV) intake in older adults – is important for designing successful interventions for promoting health and wellbeing.

Thailand is among several countries which have already become an “aged society” [15]. In 2017, approximately 17% of Thais were age 60 years or older [16]. The proportion of the population that is older adults is expected to increase to at least 20 and 28% by 2021 and 2031, respectively [16]. Due to this demographic transition, along with socioeconomic change [17], the pattern of Thai households has become more diverse, characterized by a general downsizing from large households in which aging parents and their children (and grandchildren) typically live together or in close proximity, to smaller arrangements such as skipped-generation, older adult-only, and one-person households. These changes are a direct effect of fertility decline and migration of adult children, especially from rural to urban areas [18–20]. In Thailand, the number of skipped-generation households (i.e., where the grandparent lives with one or more grandchildren but absent the grandchild’s parents) increased nearly four-fold, from 107,494 in 1987 to 405,615 in 2013. Of these, more than half were headed by person(s) age 60 years or older. About 90% of the households had a female grandparent who was raising her grandchild (ren) alone. Inevitably, this deviation from the traditional, extended family structure has the potential to adversely impact the household’s health-related behaviors, and especially FV consumption by older adults.

High prevalence rate of low FV intake among Thai older adults was observed. About two-thirds (67.9%) of the Thai population age 60 years or older had low FV intake [21]. They consumed less than 400 g per day which is considered to be insufficient according to WHO recommendations (at least five servings or 400 g per day) [22]. Sufficient FV consumption -- particularly in older adults -- can prevent or delay the onset of chronic diseases, adverse geriatric conditions, and functional impairment [23]. To improve good nutrition and promote the health and wellbeing of older adults, government policy must take into account a diversity of those demographic and socioeconomic challenges, including nutrition. A better understanding of the relationship between older adults’ diets and diverse household type is needed to guide government policy and action. This exploration will also advance academic knowledge and clinical practice, e.g., clinicians should be more sensitive to older adults’ social support and nutrition.

The association between living arrangements and older adults’ food intake -- especially FV consumption -- has been little studied. Thus, in order to fill this gap, this study investigated the association between living arrangements and FV consumption in older Thais using data from a nationally-representative sample of households.

## Methods

### Data collection and subjects

This study analyzed data from a national household survey that has been published elsewhere [21]. The survey was carried out during June–December 2018. A multistage sampling technique was used to select enumeration areas (EA), households and participants at each of the selected households. The first stage involved a systematic sample of two provinces, within each geographic region. Then within each province, districts were sampled and, from these, EA and households were selected as sampling units. All the household members who were present were recruited to participate in this study. Details of the sampling technique are described in another study [21]. The survey included a representative sample of 2048 Thais age 60 years or older. The data include FV intake, living arrangements, various health-related topics, and socio-demographic characteristics.

### Selection of variables

In this study, FV consumption was used as the outcome variable and as a proxy for healthy diet. A participant’s FV consumption was assessed based on the frequency (number of days) that the participant consumed FV in a typical week, and quantity (number of servings) consumed on each day. Servings were determined based on response to pictures of raw and cooked FV items in one serving size, adapted from the ‘Healthy Eating Guidelines’ of the Bureau of Nutrition, Department of Health, Ministry of Public Health, Thailand [24]. Details of the standard serving for FV are described in another study [21]. Daily FV consumption was categorized into two groups according to WHO recommendations: ‘less than 400 grams,’ and ‘400 grams or above.’

Living arrangements and number of household members were used as independent variables. ‘Living arrangement’ refers to the structure and composition of the respondent’s household, including the number of household members and their relationship to each another [25].

Living arrangement of the older person was categorized into five groups based on previous research [26], as shown in Table 1. The household size was recorded by asking the respondents about the total number of person(s) usually living together in the same household.

**Table 1:** Classification of living arrangement in this study

Category	Description
(a) at least one child	Living with child(ren) only, or living with child(ren) and other members, i.e., spouse, grandchild(ren), relatives and/or non-relatives.
(b) spouse only and no children	Living with no one else except spouse.
(c) at least one grandchild and no children (skipped generation)	Living with grandchild(ren) only, or living with grandchild(ren) and other members, i.e., spouse, relatives and/or non-relatives. All these cases are living without children.
(d) other relatives and/or non-relatives, and no children	Living with relatives only, with non-relatives only, with relatives and non-relatives only, or with spouse and relatives and/or non-relatives. All these cases are living without children.
(e) living alone	Living at home without anyone else.

Other variables were also included in the analysis such as socio-demographic characteristics, self-rated health status, perceived knowledge of FV, and exposure to FV promotion campaigns in the home community.

Socio-demographic characteristics include sex, age, marital status, place of residence, educational attainment, occupation, and personal income. Sex was included as a dichotomous variable; i.e., male or female. Marital status was classified into three groups: single, married, and widowed/divorced/separated. Age was categorized into four groups: 60–64, 60–69, 70–79, and 80 or above. Place of residence was dichotomized into urban or rural. Educational attainment was classified into no formal education, primary education, secondary education, and bachelor’s degree or higher. Occupation was grouped into unemployed, farmer, and non-farmer. Income (baht per month) was classified into no income, less than 10,000, 10,000–19,999 and 20,000 or above.

Self-rated health status was measured by asking participants the question ‘*How was your health in general during the past month?’* Response options were very good, good, moderate, not good, and bad. The participants were also asked to rate their level of knowledge of FV and level of exposure to FV promotion campaigns in the community. Response was categorized as highest, high, low, and lowest level.

### Statistical analysis

Sample weighting was conducted to compensate for unequal selection probabilities, for nonresponse, and calibrated to the older population counts before the data analysis. The weighting procedures were based on the weighting procedures developed for household surveys by the NSO. In this study, the sampling weight was normalized so that the distribution of sample was the same as that of weighted data. Descriptive statistics were used in describing living arrangements and FV intake, and the χ2 test was used to examine the association between FV intake, living arrangements, and each of the other independent variables. The likelihood of having sufficient FV intake was determined by binary logistic regression analysis. Binary regression analysis was used to examine the association between each independent variable with FV intake. Odds ratios (ORs) were calculated, and estimates are presented with a 95% confidence interval (CI). The ORs represent the probability of having sufficient FV intake. Any observed relationship with a *p* value of 0.05 or less (2-tailed) is considered statistically significant.

## Results

Of the 2048 participants**,** one-fourth had two household members. More than half (55.9%) of the sample reported living with at least one child, followed by living with spouse only (17.7%) and living alone (6.3%) (Table 2). The mean age of the participants was 68.2 ± 6.5 years. The mean number of persons living together in one household was 3.8. Statistically-significant differences were found in relation to all independent variables, except age and marital status.

**Table 2:** Socio-demographic characteristics of older adults, and association with FV intake

Variables	N	%Total	FV grams/day		***P*** value
			< 400	≥ 400	
**Total**	**2048**	**100.0**	**68.1**	**31.9**	–
**Sex**					
Male	918	44.8	70.6	29.4	0.030*
Female	1130	55.2	66.1	33.9	
**Age in years** (Mean = 68.2, Median = 67, SD = 6.5, Min = 60, Max = 93)					
60–64	698	34.1	67.6	32.5	0.100
65–69	593	28.9	65.8	34.2	
70–79	617	30.1	69.0	31.1	
80 or over	139	6.8	77.0	23.7	
**Place of residence**					
Urban	847	41.4	64.2	35.8	0.002**
Rural	1201	58.6	70.8	29.2	
**Marital status**					
Single	82	4.0	57.3	41.5	0.104
Married	1412	69.0	68.1	32.0	
Widowed/divorced/separated	554	27.0	69.7	30.1	
**Educational attainment**					
No formal education	322	15.7	72.7	27.3	0.009**
Primary school	1407	68.7	68.4	31.6	
Secondary school	218	10.6	64.2	35.3	
Bachelor’s or higher degree	102	5.0	55.9	44.1	
**Occupation**					
Unemployed	1163	56.8	70.7	29.3	0.005**
Farmer	429	21.0	62.2	37.8	
Non-farmer	456	22.3	66.9	33.1	
**Personal income (baht per month)**					
No income	377	18.4	76.1	23.9	0.001**
Less than 10,000	1345	65.7	67.4	32.6	
10,000-19,999	202	9.9	59.9	39.6	
20,000 or above	123	6.0	63.4	36.6	
**Self-rated health**					
Good/very good	746	36.5	64.9	35.3	0.016*
Moderate/not good/bad	1301	63.5	69.9	30.0	
**Knowledge of FV**					
High/highest	1151	56.2	64.4	35.7	0.000***
Low/lowest	897	43.8	72.8	27.1	
**Exposure to a community campaign**					
High/highest	773	37.7	63.4	36.6	0.000***
Low/lowest	1275	62.3	71.0	29.0	
**Household size (persons)** (Mean = 3.8, Median = 3, SD = 1.9, Min 1, Max 11)					
1–2	637	31.1	62.0	38.0	0.000***
3–4	745	36.4	67.8	32.2	
5 or more	665	32.5	74.3	25.9	
**Living arrangements**					
With at least one child	1144	55.9	70.0	30.0	0.001**
With spouse only	362	17.7	59.7	40.3	
Skipped-generation household	273	13.3	69.6	30.4	
With relatives or/and non- relatives	140	6.8	63.6	36.4	
Alone	129	6.3	76.7	24.0	

Note(s): *Sig. ≤ 0.05, **Sig ≤ 0.01, ***Sig ≤ 0.001

Binary logistic regression analysis was applied to assess the association between various socio-demographic factors, living arrangement, and FV intake (Table 3). The analysis found that the group with the highest probability of having sufficient FV intake was older adults with co-residential living arrangements. Those older adults who lived with at least one child or lived in a skipped-generation household were 2.7 and 2.2 times as likely to have sufficient FV intake as those who lived alone (*p* < 0.001 and *p* < 0.01, respectively). Those who lived with their spouse only were 2.1 times as likely to have sufficient FV intake as those who lived alone (*p* < 0.01).

**Table 3:** Binary logistic regression of socio-demographic characteristics and living arrangement in association with sufficient FV intake among persons age 60 years or over (*N* = 2048)

Variables	Adjusted OR (95% CI)
**Sex (Reference group = Male)**	
Female	1.491 (1.193–1.864)***
**Age (years) (Reference group = 80 or over)**	
60–64	1.112 (0.700–1.768)
65–69	1.250 (0.792–1.973)
70–79	1.226 (0.785–1.915)
**Marital Status (Reference group = Single)**	
Married	0.715 (0.385–1.327)
Widowed/divorced/separated	0.646 (0.352–1.188)
**Place of residence (Reference group = Rural)**	
Urban	1.355 (1.107–1.659)**
**Educational attainment (Reference group = No education)**	
Primary	1.282 (0.964–1.705)
Secondary	1.420 (0.946–2.131)
Bachelor’s or higher degree	1.925 (1.103–3.360)*
**Occupation (Reference group = Unemployed)**	
Farmer	1.498 (1.141–1.967)**
Non-farmer	1.032 (0.787–1.352)
**Personal income (baht per month) (Reference group = no income)**	
Below 10,000	1.416 (1.065–1.884)*
10,000-19,999	1.684 (1.113–2.546)*
20,000 or higher	1.260 (0.753–2.106)
**Household size (Reference group = 1–2)**	
3–4	0.600 (0.400–0.898)*
5 or over	0.406 (0.265–0.621)***
**Self-rated health (Reference group = Moderate/not good/bad)**	
Good/very good	1.240 (1.009–1.523)*
**Knowledge of FV (Reference group = Low/lowest)**	
High/highest	1.409 (1.151–1.726)**
**Exposure to a community campaign (Reference group = Low/lowest)**	
High/highest	1.373 (1.122–1.680)**
**Living arrangements (Reference group = Living alone)**	
With at least one child	2.742 (1.560–4.819)***
With spouse only	2.136 (1.270–3.591)**
Skipped-generation	2.195 (1.224–3.937)**
With relatives or/and non-relatives	2.223 (1.170–4.223)*

Note(s): Cox & Snell R square = 0.059, *Sig. ≤ 0.05, **Sig ≤ 0.01, ***Sig ≤ 0.001

This study also found a significant association of FV intake with other factors including sex, place of residence, educational attainment, occupation, income, household size, self-rated health, FV knowledge, and exposure to a community-based FV campaign. Female older adults were 1.5 times as likely as males to have sufficient FV intake (OR = 1.491, 95% CI 1.193–1.864). The lowest probability of having sufficient FV intake was among older adults from a household that there are five or more persons living together in one household (OR = 0.406; 95% CI 0.265–0.621). The participants who reported having high or highest overall FV knowledge were 1.4 times as likely to have sufficient FV intake as those who felt they had lower knowledge (OR = 1.409, 95% CI 1.151–1.726). The participants who lived in a community which had high or highest level of exposure to a FV promotion campaign were as likely to have sufficient FV intake as those who lived in a community with less exposure (OR = 1.373, 95% CI 1.122–1.680).

## Discussion

Despite the considerable attention of researchers on household living arrangements and health behavior of older adults, important gaps remain in the study of living arrangements and food consumption. This study found that sufficient FV intake of the older population in Thailand was significantly related to household factors, in particular, living arrangement and household size. Thus, there is a pressing need for more effective and efficient government support to maximize nutritional consumption among older Thais.

This study found a strong association between sufficient FV intake of older adults and co-residence with either related or unrelated household members. Compared to living alone, the older adults from co-residential households were more likely to have sufficient FV consumption. The practice of living with at least one child in older age might be explained by the bilateral family system in Thailand in which adult children, particularly daughters, are expected to provide support (including meal preparation) to older adult’s parents [19, 27]. On the other hand, the increased prevalence of skipped-generation households (in which adults leave their young children with the grandparents to raise) could be explained by Role Enhancement Theory in which the older adults feel pride in returning to the role of primary caregiver and shaper of a young child. Given the enhanced meaning to their life, these older adults may give higher priority to a healthy lifestyle, including eating FV [28, 29]. Despite the absence of children or other members of the younger generations in the household, living with an older adult’s spouse is seen as more beneficial than living alone. That is because a couple can provide mutual social support and care, including proper dietary intake [30]. Accordingly, co-residence can reinforce healthy lifestyle practices and better monitoring of eating behaviors, and thus contribute to increased FV intake. In the absence of co-residents, there is a strong need for outside interventions to ensure proper nutrition and health of older adults who live alone. It is also important for government strategies to be adaptable to different socio-demographic characteristics of family members who live with older adults. Some studies found that adult children living with their parents tend to be unmarried, unemployed, and less educated than those living independently [31] and, thus, may introduce unique stress in the household which can undermine an older parent’s health [32, 33]. This may affect older adults’ diets especially FV intake [34].

The irreversible trend toward smaller household size makes older adults’ FV intake a matter of growing concern. Nevertheless, in this study, older adults living in large households (i.e., five or more persons) were less likely to have sufficient FV intake compared to those living in smaller households. That finding is consistent with earlier studies from the developing world which found an inverse statistical relationship between household size and food quantity/quality/variety [35, 36]. Increasing household size leads to increased food expenditure, and ultimately lower dietary quantity/quality/variety [36]. In addition, households with a large number of dependents tended to have lower dietary diversity, especially among lower-income urban dwellers. While living alone may increase risk of low FV consumption among older adults, living in a large household would, in turn, put older adults at risk of having less FV consumption. It is therefore important for central and local government to implement interventions to increase access to low-cost, high-quality FV through such approaches as self-help FV kitchen gardening (associated with increased FV intake) [37]. Moreover, more government programs are needed to educate families, especially in the larger households, about the health benefits of diversified diets.

Sex plays an important role in the older person’s FV consumption. This study found that older women had a higher probability of having sufficient FV intake than older men. This might be due to a greater sense of responsibility for own health among women than men, such as being more active seekers of health-related information [38], and being more attentive to consumer products that affect their health [39]. This may encourage women to consume more FV than men. By contrast, older men may adhere to masculine norms such as self-reliance, stoicism, and the primacy of work [40, 41], and thus may view FV-eating as less masculine. Accordingly, this can be a barrier to access to and consumption of FV among older men. Thus, enhancing the woman’s role in the household should have a positive effect on eating behavior of the whole family [27]. Programs can build upon this natural advantage by supporting women’s income, enhancing a woman’s food-related knowledge and skills, providing technology for food preparation, and encouraging social networking with other women who are the nutritional leaders of the household. That said, men should also be encouraged to be role models for the younger generation in helping with meal preparation and consuming ample quantities of FV.

The study has some limitations. Self-reported data on the dependent variable (FV consumption) and the independent variables (health status, knowledge level of FV and exposure to a community campaign) are subject to recall bias and reporting errors. The data for this study came from a cross-sectional survey and, thus, cause and effect relationships cannot be asserted. Accordingly, there is a need for longitudinal data collection to investigate causal relationships between FV intake, living arrangements, and other household factors. Moreover, this study did not report on family income which is usually a direct indicator of socio-economic status. Older parents’ and children’s economic resources could be major determinants of the choice to co-reside. It was found that parents who move to live with their child were more likely to have lower household incomes than those with a child who moved back to their home [42].

The findings may have some limited application for other settings or cultures because of different family structures and relationships. In Western countries one of the distinguishing family features is the norm of neolocality, with new couples forming their own households [43], resulting in the situation where each family has its own values, and individualism is highly respected. Meanwhile, Thai families still tend to maintain a stronger tie between parents and their children, and many adult children will live with or nearby the home-family compound, even when they get married, out of respect for, and willingness to care for their parents. Moreover, future studies should examine family dynamics and negotiations between household members with regard to eating behavior. That information is important to help understand which family circumstances trigger a diet-related change in the context of diverse living arrangements. This would also shed more light on how eating behaviors and intergenerational exchange interact to affect nutrition and health of the older adults.

In today’s evolving society, it is a challenge to discretely classify the variegated living arrangements of family members. The classification scheme used for the current study generally suffices for comparison with other countries and cultures, since it broadly categorizes the most common living arrangements. The intricacies and unique scenarios of particular cultures or study purposes can be taken into account by defining specific subcategories of the overall categories of living arrangements defined in this study. This study did not capture other possible living arrangements, such as living nearby family, particularly in the case of children or younger relatives, or living in the same-sex or different-sex households. Subcategorizing the living arrangement categories in various dimensions and examining their role on FV eating behavior of the older adults are important tasks for future studies to match the pace of change of modern society.

## Conclusions

This study showed that the prevalence of sufficient FV consumption among older adults in Thailand is low. Older adults in co-residential living arrangements in particular living with at least one child were more likely to have sufficient FV intake than older adults who lived alone. Household size also had an impact on older adults’ FV consumption. Therefore, there is a strong need for interventions to ensure the nutrition and health of this vulnerable group of the population. The top priority for programs should be to promote quality nutritional behavior of older adults who live alone. This action should also take into account other associated factors such as level of FV knowledge, community-level campaigns, and socio-demographic factors. However, more longitudinal studies are needed to identify causal factors behind better or worse FV intake.

## Data Availability

The datasets generated and/or analyzed during the current study are not publicly available due to the need to protect respondents’ privacy and ethical restrictions, but are available from the corresponding author based upon on reasonable request.

## Abbreviations

FV: Fruit and vegetable
WHO: World Health Organization
